# Patterns of Visual Task-based Functional MRI Activation in Chronic Posterior Cerebral Artery Stroke Patients

**DOI:** 10.1007/s00062-023-01274-2

**Published:** 2023-03-03

**Authors:** Fatma Alkolfat, Aya Abdel Galeel, Ahmad R. Bassiouny, Hany Eldeeb, Ahmed Radwan, Yasmine A. Ashram

**Affiliations:** 1 Department of Neurology, Faculty of Medicine, Alexandria University, Alexandria, Egypt; 2 Department of Radiology, Faculty of Medicine, Alexandria University, Alexandria, Egypt; 3 Department of Biochemistry, Faculty of Science, Alexandria University, Alexandria, Egypt; 4 Department of Imaging and Pathology, Translational MRI, KU Leuven, Leuven, Belgium; 5 Leuven Brain Institute (LBI), Department of Neurosciences, KU Leuven, Leuven, Belgium; 6 Department of Physiology, Faculty of Medicine, Alexandria University, Alexandria, Egypt

**Keywords:** Visual stroke, Stroke recovery, Functional reorganisation, Brain remodelling, Visual perception

## Abstract

**Purpose:**

Stroke is a principal cause of disability worldwide. In motor stroke, the tools for stratification and prognostication are plentiful. Conversely, in stroke causing mainly visual and cognitive problems, there is still no gold standard modality to use. The purpose of this study was to explore the fMRI recruitment pattern in chronic posterior cerebral artery (PCA) stroke patients and to investigate fMRI as a biomarker of disability in these patients.

**Methods:**

The study included 10 chronic PCA stroke patients and another 10 age-matched volunteer controls. The clinical presentation, cognitive state, and performance in visual perceptual skills battery (TVPS-3) were determined for both patients and control groups. Task-based fMRI scans were acquired while performing a passive visual task. Individual and group analyses of the fMRI scans as well as correlation analysis with the clinical and behavioral data were done.

**Results:**

At the level of behavioral assessment there was non-selective global impairment in all visual skills subtests. On visual task-based fMRI, patients recruited more brain areas than controls. These activations were present in the ipsilesional side distributed in the ipsilesional cerebellum, dorsolateral prefrontal cortex mainly Brodmann area (BA) 9, superior parietal lobule (somatosensory associative cortex, BA 7), superior temporal gyrus (BA 22), supramarginal gyrus (BA 40), and contralesional associative visual cortex (BA 19). Spearman’s rank correlation was computed to assess the relationship between the TVPS scores and the numbers of fMRI neuronal clusters in each patient above the main control activations, there was a negative correlation between the two variables, r(10) = −0.85, *p* ≤ 0.001.

**Conclusion:**

In chronic PCA stroke patients with residual visual impairments, the brain attempts to recruit more neighboring and distant functional areas for executing the impaired visual skill. This intense recruitment pattern in poorly recovering patients appears to be a sign of failed compensation. Consequently, fMRI has the potential for clinically relevant prognostic assessment in patients surviving PCA stroke; however, as this study included no longitudinal data, this potential should be further investigated in longitudinal imaging studies, with a larger cohort, and multiple time points.

## Introduction

Cerebrovascular diseases remain the second leading cause of disability and mortality worldwide [1]. While most attention has been focused on its motor sequelae, the umbrella of stroke disabilities encompasses a wider range of less tangible deficits affecting cognitive, affective, and visual functions [2]. Posterior cerebral artery (PCA) stroke represents about 5–10% of all strokes [3]. The PCA supplies the occipital and inferior medial temporal cortical areas as well as deeper regions of the thalamus and the mid-brain [3, 4]. As a result, the clinical picture of PCA stroke involves a wide spectrum of syndromes according to the locality of vascular occlusion and the extent of the collateral circulation [5]. It includes mainly visual field defects such as contralateral homonymous hemianopia, or quadrantanopia; contralateral limb numbness and weakness, cognitive impairment and various more visual perceptual dysfunctions [6, 7].

The only effective treatment for stroke requires intervention during the hyperacute phase for reperfusion of ischemic brain areas prior to irreversible cell death. Unfortunately, a substantial number of patients do not get treated within this golden window and inevitably develop chronic sequelae for which the only treatment is neurophysical rehabilitation. While there are clearly defined rehabilitation strategies for patients with sensory motor deficits, the less fortunate patients with visual disabilities are usually overlooked. The journey of rehabilitation starts with a better understanding of visual and cognitive deficits, stratification of the deficits, as well as prognostication. In the current status quo, we have some tools in our hands, starting from clinical scores and neuropsychological batteries and ending with more advanced neuroimaging. For clinical scores, the simple and famous clinical disability ones, such as the modified Rankin Scale (mRS), are optimal for the classical phenotype of stroke disability, the mobility one, underestimating the functional consequences of visual deficit strokes [8]. Neuropsychological batteries are useful clinical measures, but there is no consensus on the batteries to be used in stroke patients [9]. Besides, they are time-consuming; need a certain level of patient cooperation, and skilled personnel who are not readily available in less developed countries. Structural neuroimaging is the main investigation used by stroke clinicians; however, it does not reflect the certainly changing levels of functional performance and organization throughout the course of stroke. Therefore, the need for other ways to objectively reveal the level of disability is undeniable. The aspects of functional MRI (fMRI), such as its dynamic, non-invasive, and objective nature, make it attractive for exploring its applicability in stroke patients. Both resting and task-based modalities of fMRI of the brain are being increasingly utilized in stroke patients to assess and predict recovery [10]. Despite the different methodologies and diverse results among studies, certain patterns of change were detected [11–14]. Previous studies using fMRI focused mainly on motor and speech areas [15–19]. In general, results revealed that the brain recruits more ipsilesional and contralesional networks to do the impaired task of the infarcted areas in the early phase. In time, the more optimal the recovery is, the fewer networks are demanded to be recruited and the brain shifts to near-normal focused pathways of activation [15, 16, 20–22].

There are still unanswered questions regarding the association between the real visual performance of patients and the pattern of visual task-based fMRI activations in PCA stroke patients. In this study, we aimed to explore the fMRI ipsilesional and contralesional recruitment pattern when the primary visual areas were partly affected by the stroke, and to investigate fMRI as a marker of disability in stroke affecting visual functions.

## Material and Methods

### Study Design and Patient Population

This prospective patient-control study included 10 patients with radiological evidence (CT or MRI brain) of a stroke involving the cortical territory of the PCA. All patients were undergoing clinical follow-up at the stroke clinic of the Neurology Department in our tertiary referral center and 10 healthy volunteers with a similar age range and level of education were also recruited as a control group. These volunteers had no history of drug abuse, neurological, psychiatric, or ophthalmological disorders. The study was conducted in accordance with the World Medical Association (WMA) declaration of Helsinki, 1964, and after the approval of the institutional ethical committee. Verbal consent was received from all patients and control.

Patients were included in this study if they were diagnosed with a PCA stroke on an imaging (CT and/or MRI) and clinical basis, at least 3 months before the study scan date, and were older than 18 years of age. Patients were excluded if they had other sizable or clinically significant brain lesions, claustrophobia, severe cognitive impairment interfering with the understanding of the task instructions, alcoholism and/or drug addiction, and/or if there was a moderate to severe visual impairment.

### Clinical and Behavioral Assessment

All studied patients were subjected to complete history taking and complete physical and neurological examination. In addition, a detailed ophthalmological assessment including visual acuity and visual field perimetry was performed for all patients and controls. The mini-mental state examination (MMSE) [23] was used as a measure of cognitive state stratification, and the test of visual perceptual skills-third edition (TVPS-3) were done to assess visual perception [24–26]. The TVPS‑3 battery utilizes black and white line drawings as stimuli for all the perceptual tasks. The items were presented in a multiple-choice format, and responses were either verbal or by pointing to the answer choice. This format was ideal for use with subjects having impairments in motor, speech, hearing, neurological, or cognitive functions. There were 16 question cards in each of the perceptual subsets: visual discrimination, visuospatial relationships, visual memory, visual sequential memory, form constancy, figure-ground perception and visual closure.

### MRI Acquisition

Data were acquired with a 3T Philips (MRI) scanner using a 32-channel head coil. All functional acquisitions used a gradient echo, echo planar sequence with a 160 square matrix (slice thickness of 4 mm, interleaved acquisition) leading to an in-plane resolution of 2.4 × 2.44 mm^2^, 34 slices per volume, the total number of volumes per MRI run *N* = 110, repetition time (TR) = 3 s, echo time (TE) = 35 ms and flip angle = 90°. The total duration of the functional scan acquisition was 5 min and 40 s. High-resolution structural scans were acquired in each scan session for registration to surface anatomical images; matrix = 256; [TR = 1.9 s; TE = 3.9 ms; flip angle = 9°, 1 × 1 × 1 mm^3^ resolution].

### Visual Paradigm used in fMRI

**Fig. 1:**
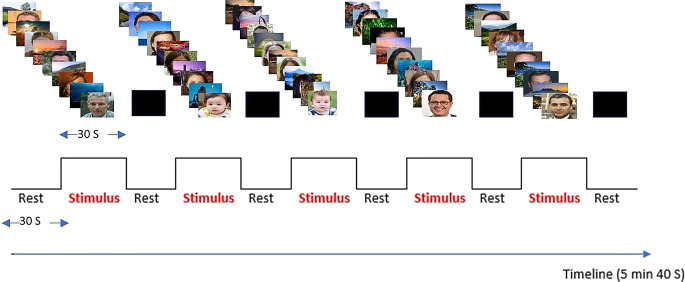
TimeLine demonstrates the visual paradigm displayed during functional MRI acquisition

A symmetrical block design was used consisting of 11 epochs of 30‑s each alternating between rest and task performance. During each 30- s epoch 10 scans were acquired so the total number of functional scans was 110 scans per run (Fig. 1). The patients and control subjects were instructed to passively look at the displayed images/black screens that were reflected on the small mirrors attached to the head coil. During rest periods, absolute darkness, i.e., a black screen was presented. During the visual stimulation, alternating images of faces and landscapes were displayed. The faces were of fictitious persons developed through artificial intelligence and were accessed through the *website: thispersondoesnotexist.com*. The landscape pictures were accessed from online windows landscape wallpapers. Visual stimuli were presented using a slide show that was timed and synchronized to the fMRI paradigm. A projector placed in the console room was used to project the slide show onto an MRI-compatible screen placed at the foot of the MRI table and visible to the patients through mirrors mounted on the front of the head coil.

### Lesion Mapping

Structural images (T1 scans) were spatially normalized using Statistical Parametric Mapping software SPM12 (Welcome Trust Centre for Neuroimaging, Institute of Neurology, University College London, UK) running under Matlab R2020a (The Mathworks, Inc., Natick, MA, USA). Individual lesions were defined based on visible damage and hypointensities on T1 images. The lesions were semi-automatically delineated through MRIcron. Lesions were then displayed against the Brodmann atlas within the MRIcron template library. The total number of voxels and the number of lesion voxels per each Brodmann area were reported per patient. The images in Neuroimaging Informatics Technology Initiative format (nifti) of lesions were used as an exclusive mask per individual in first-level analysis.

### Analysis of fMRI

The fMRI analysis was performed using SPM12. Structural and functional scans from patients with left-sided PCA were flipped before preprocessing so that the right side was always the ipsilesional one.

#### Preprocessing

This stage included slice timing, motion correction (realign), co-registering (coregister) to match the fMRI scans with the anatomical scans, spatial normalization (normalize) of the fMRI, and anatomical scans in relation to Montreal Neurological Institute (MNI) template space and finally, spatial smoothing of fMRI images using a 3D Gaussian smoothing kernel with an 8 mm at full width at half maximum. Realignment graphs of image translation and rotation were reviewed. Participants were excluded if their maximum displacement reached 2 mm in the X, Y, and Z axes and 2.5° in the angular rotation around each axis.

#### First-level Analysis

The data were then subjected to statistical analysis using the general linear model (GLM). This included the following steps: first, specification of the GLM design matrix by entering various timing parameters, fMRI scans and conditions (a vector of onset times, event durations, orthogonalize modulations), and motion parameters as multiple regressors, filtering (high-pass filter with a cut-off of 128 s), masking threshold (defaults value of 0.8) and serial correlations using an autoregressive AR (1) model. Then estimation of GLM parameters using restricted maximum likelihood (ReML), interrogation of results using contrast vectors, and application of exclusive lesion mask per individual to produce statistical parametric maps (SPMs). The statistical significance threshold of *p*-value < 0.001 was provided, uncorrected to family-wise error (FWE) due to the small sample size. The GLM of all participants was done, then t‑test statistics comparing the conditions of rest (absolute darkness) and visual stimuli (seeing pictures) in each run to generate an SPM for each run in the first-level analysis.

#### Second-level Analysis

The first-level analysis data of all participants were fed into the second-level analysis to compare the two groups. A two-sample t‑test using SPM 12 GUI was done to compare the patient group and the control group. The difference between the two groups was explored through the application of two contrasts; the first one is patient-control contrast to elicit significant activations in the patient group that were not present in the control group. The second one is control-patient contrast to elicit the significant activations that were only present in the control group. A third comparison was done between individual SPM (that resulted from the first-level analysis) and the mean control SPM using the contrast (patient-control). This was to elicit the significant clusters that were exclusively present in every single patient in comparison to the mean SPM of the control group. The SPMs of the second-level analysis were generated at the threshold of *p*-value < 0.001, uncorrected to FWE with a cluster size of 30 voxels.

### Statistical Analysis

Data were fed to the computer and analyzed using IBM SPSS software package version 20.0*. *(IBM Corp, Armonk, NY, USA). The Kolmogorov-Smirnov test was used to verify the normality of distribution. Quantitative data were described using range (minimum and maximum), mean, standard deviation, and median. An independent sample t‑test was done to compare clinical scores between cases and control. Spearman and Pearson correlation analyses were done to elicit correlations between the radiological variables and patients’ performance at the TVPS test. The significance of the obtained results was judged at least at the 5% level.

## Results

### Clinical Data

In this study 10 patients in the chronic phase of an ischemic posterior cerebral artery stroke comprising 7 males and 3 females were recruited. All patients were right-handed, and their ages ranged between 47 and 70 years (mean 58.6 ± 7.3 years). At the time of assessment, 3 months minimum and 28 months maximum had passed since the onset of the stroke (mean 13.6 ± 9.3 months). Patient assessments for the presence of stroke risk factors revealed hypertension to be the most common factor in this sample as it existed in 8 patients. This was followed by smoking, diabetes mellitus, ischemic heart diseases (IHD), valvular heart disease, and atrial fibrillation each in one patient. Regarding the clinical presentation of the stroke, all patients had visual problems, however, only 5 of them could define it as a field problem while the other 5 stated they had only blurry vision, 8 patients had mild contralateral limb weakness, various degrees of contralateral numbness, and disturbing paresthesia. Half of the patients had a history of confusion in the acute phase, that recovered with various degrees of residual cognitive impairment. Patient demographic and clinical characteristics are listed in (Table 1). A total of 10 right-handed control volunteers, 6 males and 4 females, aged between 45 and 63 years (mean 53 ± 7.3 years) were enrolled in this study (Table 2). Their best corrected visual acuity was 6/6, and they had no history of neurological, psychiatric, or substance misuse disorders.

**Table 1:** Demographic and clinical characteristics of the stroke patients (*n* = 10)

	Age (years)	Gender	Level of education	Time since stroke (months)	Stroke side	Risk factors	Clinical presentation
Patient 1	63	M	Primary	4	R	DM, HTN, Smoking	Contralateral weakness, numbness, visual problem
Patient 2	70	F	Primary	10	L	DM, HTN, IHD	Visual problem
Patient 3	62	M	Primary	24	R	DM, HTN, Smoking, IHD	Contralateral weakness, numbness, visual problem
Patient 4	47	M	Intermediate	21	R	DM, HTN, Smoking	Visual problem, cognitive impairment
Patient 5	61	M	Primary	7	L	Smoking	Contralateral weakness, numbness, visual problem
Patient 6	53	M	Intermediate	28	R	HTN	Contralateral weakness, numbness, visual problem, cognitive impairment
Patient 7	51	F	Primary	23	L	HTN, valvular heart disease	Contralateral weakness, visual problem
Patient 8	61	M	Illiterate	9	L	DM, HTN, Smoking, IHD	Contralateral weakness, numbness, visual problem, cognitive impairment
Patient 9	65	F	Primary	3	L	DM, HTN, AF, IHD	Contralateral weakness, numbness, visual problem, cognitive impairment
Patient 10	53	M	Intermediate	7	L	Smoking	Contralateral weakness, numbness, visual problem, cognitive impairment

*M* male, *F* female, *R* right, *L* left, *DM* diabetes mellitus, *HTN* hypertension, *IHD* ischemic heart disease, *AF* atrial fibrillation

**Table 2:** Demographic and clinical characteristics of the control subjects

	Age (years)	Gender	Level of education	Comorbidities and habits
Control 1	45	M	Intermediate	–
Control 2	63	M	Intermediate	HTN, IHD, Smoking
Control 3	54	M	Intermediate	Smoking
Control 4	57	F	Intermediate	HTN
Control 5	48	F	High	Asthma
Control 6	55	M	Primary	–
Control 7	58	M	Primary	HTN
Control 8	54	F	Primary	DM
Control 9	45	F	Intermediate	–
Control 10	55	M	Intermediate	Smoking

*M* male, *F* female, *DM* diabetes mellitus, *HTN* hypertension, *IHD* ischemic heart disease

### Behavioral Data

Cognitive impairment was evaluated through MMSE; 4 patients had no cognitive impairment (MMSE ≥ 25), 2 patients had mild impairment (MMSE 21–24), and 4 patients had moderate impairment (MMSE 10–20) [23].

The test of visual perceptual skills 3 (TVPS-3) was done for both cases and controls and patients demonstrated statistically significant lower performance across all subtests categories (patient group, *n* = 10, mean = 6.45 ± 4.45) compared to the control group (control group, *n* = 10, mean = 11.93 ± 1.08), t(18) = 3.5, *P* =0.02. Patients performed best at the visual discrimination subtest TVPS-VD (mean = 8.25 ± 5.3) and worst at visual closure subtest (mean = 4.9 ± 4.1). The greatest difference between patients and the control group was in the visuospatial relationships TVPS-VSR subtest (mean = 7.3 ± 1.57).

### MRI Results

#### Structural MRI Lesion Mapping

**Fig. 2:**
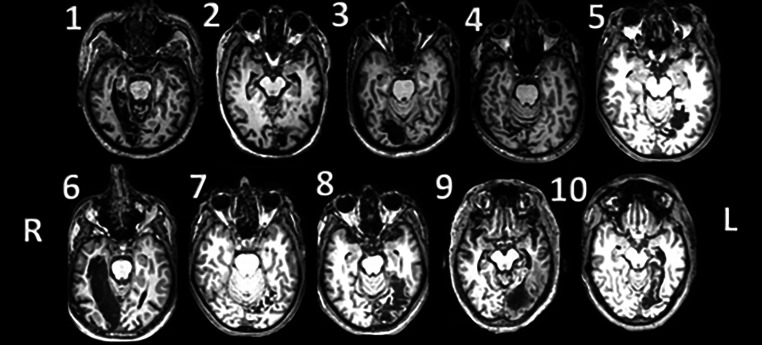
Axial structural T1-weighted MRI brain scans at the level of maximum infarct volume for each patient performed at the time of the fMRI acquisition

**Fig. 3:**
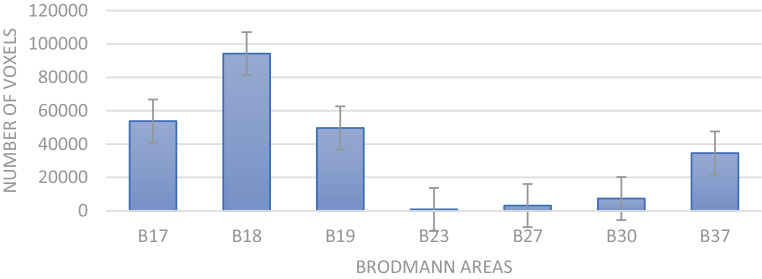
Graph showing the contribution of each Brodmann area to the structural infarction volume in all stroke patients. *Blocks* represent volumes of the broadmann areas in voxels, while *error bars* are measurement variability generated by the mapping software

Of the patients six had a stroke on the left side while four were on the right side. The site of cerebral infarction was determined from the T1-weighted structural MRI (Fig. 2). The lesions were mapped using MRICron, and lesion maps were plotted against the Brodmann atlas to draw statistics regarding the anatomical distribution of the lesion volumes in voxels. Brodmann areas 18, 17, 19, and 37 were the most predominantly affected areas in this sample of PCA stroke patients (Fig. 3).

#### Results of fMRI

##### First-level Analysis: SPMs of Individual Patients and Controls:

**Fig. 4:**
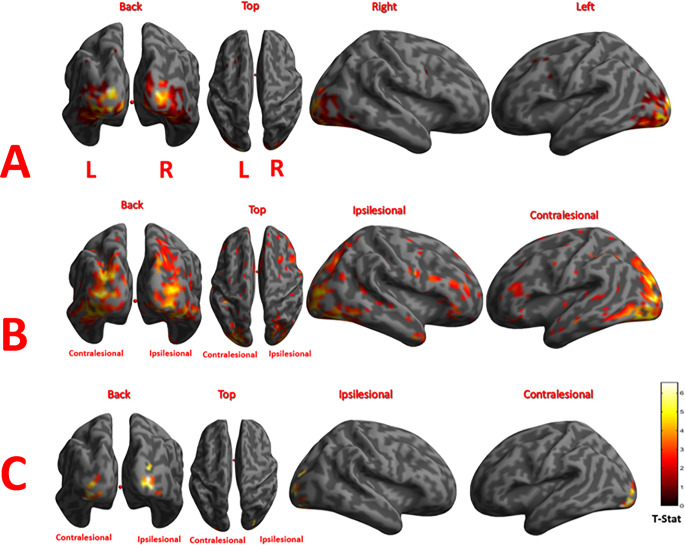
The first-level statistically significant activation in the state of seeing over the resting state in one of the control subjects (A), patient-2 (B), and patient-4 (C) rendered on the surface brain. Different views of the 3D brain surface show the activated regions. The color bar is coding for the T‑Stat score

Here, examples are shown for one control, one patient with good recovery, and one patient with poor recovery. An SPM of a 63-year-old healthy control showed significant activations in the visual occipital areas (Fig. 4-A). Another SPM of a 70-year-old patient (patient 2) with a history of left PCA stroke which resulted in mild cognitive impairment and poor performance in the visual skills tests. The activations in this SPM were dispersed over temporal, frontal, and parietal lobes (Fig. 4-B). Lastly, an SPM of a 47-year-old patient (patient 4) with a history of right PCA stroke. This patient had no cognitive impairment and good performance in the visual skills tests. The activations in his SPM were only present in the occipital lobes as demonstrated in (Fig. 4-C).

##### Second-level Analysis (Group Analysis): Comparison Between the Control Group and Patient Group:

**Fig. 5:**
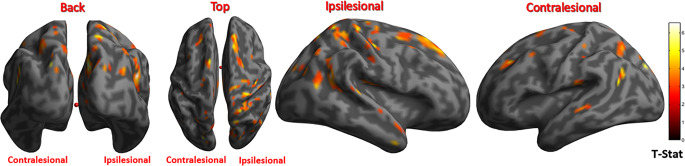
Different views for the surface-rendered cortical activations that are present in stroke patients in a higher intensity than the control after second level (group) analysis. Note that the clusters are more on the ipsilesional side, and involve frontal, parietal, and temporal lobes. The color bar is coding for the T‑score

i Task-based activation comparison between patients and controls:

The comparison between the group of patients and controls using (patient-control) contrast produced nine clusters of activations that were significantly present in the patient group, eight of which were ipsilesional in the frontal, parietal, and temporal lobes. The exact anatomical sites are demonstrated in (Table 3). The clusters are superimposed on a 3D brain surface with back, top, right, and left views (Fig. 5).

**Table 3:** MNI coordinates of peak intensities and anatomical structures involved in the Clusters of activation in cases compared to controls at *p* < 0.001 and cluster size: 30 voxels. The right side (R) is the ipsilesional site

Clusters	Number of voxels	Peak MNI coordinate			Structures	Brodmann area	T‑score	*P*-value
		X	Y	Z				
Cluster 1	51	20	−42	−44	R cerebellum/tonsil	–	5.17	< 0.001
Cluster 2	87	2	−66	−4	R cerebellum	–	4.74	< 0.001
Cluster 3	106	50	−54	24	R superior temporal gyrus	BA 22	5.71	< 0.001
Cluster 4	254	6	−68	30	R cuneus/precuneus	BA 7	6.12	< 0.001
Cluster 5	35	−22	−84	34	L parietal lobe/precuneus	BA 19	6.06	< 0.001
Cluster 6	35	18	32	44	R frontal lobe	BA 9	5.09	< 0.001
Cluster 7	32	22	−44	52	R parietal lobe	BA 40	4,78	< 0.001
Cluster 8	167	34	−48	66	R parietal lobe	BA 7	7.83	< 0.001
Cluster 9	34	12	−26	72	R frontal lobe	BA 4	5.00	< 0.001

*L* left, *R* right, *BA* Brodmann area, *MNI* Montreal Neurological Institute

**Fig. 6:**
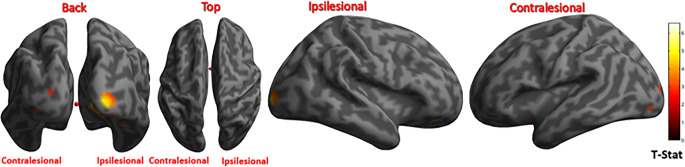
Different views for the surface-rendered cortical activations that are present in control subjects in a higher intensity than patients after second level (group) analysis. Note that the clusters are mainly occipital, on the ipsilesional side. The color bar is coding for the T‑score

Another comparison was done between the group of patients and controls using the contrast (Control-Patient) and produced 4 clusters that were exclusively present in the control group. Their anatomical sites are demonstrated in (Table 4). The clusters are superimposed on a 3D brain surface with back, top, right, and left views (Fig. 6).

ii Comparison between every single patient and control group and correlation with functional performance:

**Table 4:** Peak MNI coordinates, and anatomical structures involved in the clusters of activation in controls compared to patients at *p* < 0.001 and cluster size: 30 voxels. The right side (R) is the ipsilesional side

Cluster	Size in voxels	Peak MNI coordinate			Structures	Brodmann area	T‑score	*P*-value
		X	Y	Z				
**Cluster 1**	38	−8	−46	−30	L Cerebellum	—	5.15	< 0.001
**Cluster 2**	33	40	−72	−26	R Cerebellum	—	4.63	< 0.001
**Cluster 3**	183	14	−96	0	R Middle occipital	BA 17, 18	5.79	< 0.001
**Cluster 4**	78	−24	−86	2	R Cuneus	BA 18, 19	5.55	< 0.001

*L* left, *R* right, *BA* Brodmann area, *MNI* Montreal Neurological Institute

The clusters of activations in every single patient that were not present or activated at the same intensity in the control group (mean of the group) were drawn for every patient. Ranking the patients by visual performance score (TVPS scores) poorer outcomes towards the left, better outcomes towards the right, and plotting the clusters according to their anatomical distribution have shown that most of the clusters were crowded toward the left (the poorer outcome). The most frequently reported lobes were the ipsilesional frontal followed by parietal and temporal lobes (Table 5). Furthermore, these patterns were statistically explored through the calculation of the Spearman’s correlation coefficients between the number of fMRI clusters in each case over the mean control and other variables including TVPS score, MMSE, and the total lesion size in voxels (Table 6).

**Table 5:** Task-related activity for stroke patients compared with the control group. Each (+) represents a cluster with a minimum size of 30 voxels. Patients are ranked according to the total TVPS score. Patients ranked by visual performance score (poorer outcome towards the left, better outcome towards the right)

		Patient‑8	Patient-10	Patient‑9	Patient‑2	Patient‑3	Patient‑1	Patient‑7	Patient‑4	Patient‑5	Patient‑6
Clusters number		5	27	17	9	10	2	0	0	0	1
Frontal lobe	IL	++	++++	++++++	+++	++	+	–	–	–	–
	CL	–	+++	+	+	++	–	–	–	–	–
Parietal lobe	IL	–	++++	+++	–	++	–	–	–	–	–
	CL	+	+++	–	–	–	–	–	–	–	+
Temporal lobe	IL	–	+++++	+	+	+	–	–	–	–	–
	CL	+	+	++	+	+	+	–	–	–	–
Occipital lobe	IL	–	+	+	–	+	–	–	–	–	–
	CL	–	–	–	+	–	–	–	–	–	–
Basal ganglia	IL	–	+	–	–	–	–	–	–	–	–
	CL	–	+	+	–	+	–	–	–	–	–
Cerebellum	IL	–	++	++	+	–	–	–	–	–	–
	CL	+	++	–	+	–	–	–	–	–	–

*IL* ipsilesional, *CL* contralesional, *TVPS* test of visual perceptual skills

**Table 6:** Spearman’s correlation coefficient between the number of fMRI clusters in each case over the mean control and other variables including TVPS score, MMSE, and the total lesion size in voxels

Clinical and anatomical variables	The number of fMRI clusters of activation in each case above the mean of the controls	Bivariate analysis Test of the correlation coefficient	Level of significance	Total number (*N*)
Average TVPS score	−0.855^**^	Spearman’s rho	< 0.001	*N* = 10
MMSE (cognitive state)	0.581	Spearman’s rho	0.048	
Total lesion size (in voxels)	0.384	Spearman’s rho	0.217	

*TVPS* test of visual perceptual skills, *MMSE* mini-mental state examination, *fMRI* functional magnetic resonance imaging

**Correlation is significant at the 0.01 level

Pearson correlation analysis was done to examine the correlation between TVPS scores representing the visual functional performance of the patients and other clinical variables and stroke anatomical characteristics. A strong negative correlation was found between TVPS scores in all categories and cognitive impairment level, r(9) = −0.75 [r: Pearson correlation coefficient], *p* =0.005. On the other hand, no significant correlation could be elicited between TVPS scores and the total stroke lesion volumes in voxels.

## Discussion

The main scope of this study was to explore visual tb-fMRI recruitment patterns in patients surviving cortical PCA infarctions and to investigate the utility of this method as a prognostication tool in stroke causing visual disabilities. To our knowledge, visual tb-fMRI studies have not been previously studied in these patients.

The clinical presentation spectrum of our patients included visual problems such as hemianopia, deficits in visual perceptual skills, contralateral limb weakness and numbness, and various degrees of cognitive impairment. This was in line with previous studies [5, 27, 28]. Nonetheless, cognitive impairment was overrepresented in our sample, possibly because our patients tend to seek medical attention if their symptoms were significantly disabling, and small PCA stroke tends to pass unnoticed especially if the patient is not involved in a visually demanding occupation.

In this work, the extent and categorical deficits in the visual perception pathway were evaluated using the motor-free battery of TVPS‑3 due to its reliability [24] and validity [25, 26]. In the patient group, the lesion volumes were variable as assessed by MRI. A relationship between the TVPS score and the total stroke lesion volume could not be elicited. Although this finding may appear counterintuitive, it is readily understandable in other types of stroke. For example, a small stroke in the internal capsule results in more dense weakness than a larger cortical one [29]. Similarly, patient‑6 in the present series had the largest stroke size, meanwhile, he had no cognitive impairment and scored well in the TVPS battery. He had thalamic involvement which caused contralateral severe numbness but spared the cognition. This patient was relatively young at stroke onset (53 years), had no prior brain insults, and was maintaining an acceptable functional level; factors that might explain the good functional outcome of this particular patient. Although stroke size is still an important prognostic factor, the patient functional outcome is dependent on a multitude of other factors which necessitate a holistic approach to stroke outcome.

The fMRI activation pattern in patients compared to the control group revealed a significant increase in task-related activation in several brain regions above that seen in the control group performing the same task. Most of these clusters were on the ipsilesional side, distributed in the ipsilesional cerebellum, dorsolateral prefrontal cortex (BA 9), superior parietal lobule (somatosensory associative cortex, BA 7), superior temporal gyrus (BA 22), supramarginal gyrus (BA 40), and contralesional associative visual cortex (BA 19). Explanation of these findings requires an elaboration of the functional specialization of the most significant clusters. Although the cerebellar clusters were demonstrated in both patients and control groups, they were of larger voxel size in the patient group. The cerebellum is classically associated with fine motor control; however, there is growing evidence linking the cerebellum to a multitude of cognitive functions [30]. Furthermore, it was found that portions of the cerebellum, which exhibit functional connectivity with the cortical dorsal attention network, are recruited by visual working memory and visual attention tasks [31]. This may explain why a visual task significantly activated clusters in the cerebellum, especially in the patient group.

In addition, the reason behind the recruitment of the superior parietal lobule (BA 7) in a visual task-based activation, is the role this area plays in visuomotor coordination. It is involved in the visual computation of moving objects [32]. Furthermore, it is part of the network that visually plans for hand movement i.e., grasping objects [33]. The patient group harnessed more of this associative cortex during a visual experience, hypothetically, as compensation to the infarcted primary and secondary visual cortices.

Similarly, the dorsolateral prefrontal cortex DLPFC (BA 9), was more activated in the patients’ group specifically the more disabled ones. The DLPFC has a multitude of executive functions including planning, working memory, decision making, and cognitive flexibility [34–36]. Moreover, this area represents the endpoint of the dorsal stream of the visual pathway [37]. This explains its activation in the context of visual stimulation, especially in patients who need more attention compared to controls reflecting the anticipated relative complexity of the visual task.

The superior temporal gyrus (BA 22) and the supramarginal gyrus (BA 40) are mainly auditory associative areas that play a role in speech processing. As these areas are mainly auditory associative areas, their activation upon visual stimulation in our patients is a point of interest. Cross-modal perceptual interaction between auditory and visual stimuli has been proven in several experiments [38–40].

Patients in the present study, especially those with poorer outcomes, were recruiting more circuits from the auditory association cortex. A possible reason for this increase in patients’ task-based activation clusters could be the intensified patients’ effort to perform the task relative to the controls. It is well established that the more complex the task, the more extensive the recruited neural network [41]. Another possible mechanism is neuronal reorganization or neuroplasticity; however, this mechanism could not be concluded from the present work as it is only a single-time scan, but previous longitudinal studies advocated this point as the activations appear after a few months from onset, in the setting of repeating the same task [19]; however, the significance of this finding, as a poor prognostic sign, needs further assessment in a larger cohort of patients.

The fMRI activation pattern in the control group was more focused on the Brodmann areas 17, 18, and 19 corresponding to the primary, secondary, and associative visual cortices. These areas showed a significant increase in activation when compared to the patient group on the ipsilesional side and to a lesser extent on the contralesional side. Such findings reflect the shift in peak activation in the patient group as it becomes more diffuse. This gives a hint about how the cortical regions reorganize after a focal insult. In addition, it highlights how the healthy brain tends to perform tasks more efficiently. This finding comes in agreement with previous studies on stroke affecting the motor cortex [42, 43].

In the present work, an inverse relationship between the visual perceptual scores, as a scale of the stroke residual disability, and the number of neuronal clusters activated to do the visual task in the fMRI was demonstrated. The number of clusters of activation reflects the circuits recruited to compensate for the infarcted area in doing the impaired visual task. The fMRI findings in the chronic PCA stroke replicate what is happening in stroke affecting the motor areas [42].

Although the role of contralesional recruitment has been investigated in many studies, the results were diverse and variable between the acute and chronic stroke phases. Recruitment in the early phase of PCA stroke was studied by Kim et al. using serial resting state fMRI (RS-fMRI) and visual field tests within 1 week and at 1 and 3 months after (PCA) stroke onset [49]. The interhemispheric resting state functional connectivity (RSFC) in the visual cortex within 1 week was positively correlated with the follow-up visual field tests [49]. This reflects that early recruitment of contralesional visual circuits through interhemispheric connections may be part of the process of recovery. Similarly, in the research on motor stroke, the contralesional primary motor cortex showed gradually increasing activity starting from the first 2 weeks and was correlated with improved functional recovery [50]. In addition, transcranial magnetic stimulation (TMS) studies suggested that early contralesional activation may be due to increased cortical excitability as a result of reduced inhibition over the contralesional cortex [51]. On the other hand, recruitment in the chronic phase was studied in the present work where the contralesional hemisphere was significantly activated in comparison to healthy controls mainly in BA 19. While the early contralesional activation was considered a biomarker of good recovery, the persistence of this activation in the chronic phase (beyond 6 months post-stroke) was linked to poor recovery and considered a pathological activation [46, 47, 52].

Longitudinal studies are few but invaluable in terms of interpretation of the reorganizational pathways. A longitudinal fMRI study for 1 year in 8 patients with motor stroke revealed an ipsilesional hyperactivation pattern as well as displacement of the area of the maximum activation [15]. Another longitudinal study in 10 patients with motor stroke gave insights into the transition from contralesional activation in the subacute phase into ipsilesional activation in the chronic phase [53].

Functional reorganization of cortical pathways following central or peripheral nervous lesions is now a widely explored phenomenon. While stroke is a clear example of central pathologies triggering the reorganizational pathways, it is not the only one. Multiple sclerosis (MS) was also reported to boost both healthy as well as additional pathways that are not usually activated in healthy controls [54, 55]. Although the intensity of these activations correlated with the structural T2 load [56] and were thought to be adaptive, their exact beneficial role in MS patients has yet to be studied. Interestingly, patients with cervical spinal cord compression showed increased activations compared to controls in the precentral motor cortex which increased even further 6 months after decompressive surgery [57]. Peripherally, a famous practical example of cortical reorganization in response to peripheral pathology is sensitization in chronic pain disorders. In a large-scale meta-analysis, patients with chronic pain showed functional reorganization represented in increased fMRI activations in pain-specific somatosensory cortices [58].

This study took a comprehensive approach to the patient’s functional condition. Nevertheless, there are some limitations. One limitation is the number of patients, the inclusion of a larger sample of patients would increase the statistical power of the study, and the replicability of the results as well. Additionally, in this study, we scanned the patients at a single point in time. A serial longitudinal study of the same patients starting from acute through subacute and chronic phases would help discriminate activations due to neuroplasticity rather than due to the complexity of the task for stroke patients and better understand the dynamic process of neural reorganization. Although the TVPS‑3 battery is validated and well-supported by the most recent theories of visual processing, it misses some important aspects of visual perception categorization, for example, face perception and word reading. Using multiple neuropsychological visual batteries will provide a more holistic view of the visual function status.

## Conclusion

To recapitulate, in chronic posterior cerebral artery (PCA) stroke patients with residual visual impairments, the brain attempts to recruit more neighboring and distant functional areas for executing the impaired visual skill. This intense recruitment pattern in poorly recovering patients appears to be a sign of failed compensation. Consequently, fMRI has the potential for clinically relevant prognostic assessment in patients surviving PCA stroke; however, as this study included no longitudinal data, this potential should be further investigated in longitudinal imaging studies, with a larger cohort, and multiple time points.

## Abbreviations

AF: Atrial fibrillation
AR: Autoregressive
BA: Brodmann area
CL: Contralesional
CT: Computed tomography
DLPFC: Dorsolateral prefrontal cortex
DM: Diabetes mellitus
FMRI: Functional magnetic resonance imaging
FWE: Family-wise error
GLM: General linear model
GUI: Graphical user interface
HTN: Hypertension
IHD: Ischemic heart disease
IL: Ipsilesional
MMSE: Mini-mental state examination
MNI: Montreal Neurological Institute
MS: Multiple sclerosis
Nifti: Neuroimaging Informatics Technology Initiative
PCA: Posterior cerebral artery
SPM: Statistical parametric mapping
SPSS: Statistical Package for the Social Sciences
TB-fMRI: Task-based functional magnetic resonance imaging
TE: Echo time
TMS: Transcranial magnetic stimulation
TR: Repetition time
TVPS‑3: Test of visual perceptual skills 3
WMA: World Medical Association
